# The Last Word

**Published:** 1905-12

**Authors:** 


### THE LAST WORD.

There is deep meaning to the last word. It may be one of affection, or one of anger. It may be the farewell of the departing guest, or the broken message of the dying, but whether these or more, it carries with it a feeling of loss, something given up, some chain of association broken and, above all, the world has in a measure changed in its relation to the individual and can never be exactly the same.

The man who has been year by year brought into close communion with his fellows through any public position of responsibility, probably feels the breaking of ties more acutely than others in the more loosely attached business relations. To this class the Editor belongs. His publication has been, to a greater or lesser extent, a centre of influence, and that influence will reflect upon him for good or ill in proportion as his power has been used intelligently. The subtile influence of editorship cannot be described in words. It holds, with ever-increasing attractiveness, its votaries and few voluntarily relinquish its arduous duties. There is probably no calling that financially pays so poorly and none that yields a greater compensation in that which money cannot buy,—that of bringing the individual in close touch with a world he may never see, or with which he is only indirectly in association. Yet it is this ever-revolving mysterious life that invigorates his being, renewing daily the old thought and infusing broader aspirations that his work may be better to-morrow than it has been to-day. To such a man the last word cannot be written without the sundering of many ties, the attenuated threads of which reach out and beyond the ordinary confines of a busy life.

The Editor-in-Chief of the INTERNATIONAL DENTAL JOURNAL has been the active supporter of its varied interests for the past now nearly sixteen years. He came into the service early in 1890, immediately succeeding Dr. Suddeth, who had charge the two previous years. It is unnecessary that the writer should recount his personal trials in the management of a periodical such as this. These have been many, but they can be left, like the flotsam of the world's wrecks, to float away and be forgotten. It may be said that the sixteen years have been hopeful years and at no period has the virus of discouragement entered the domain of the editorial sanctum. The INTERNATIONAL DENTAL JOURNAL was established on a principle and the struggle to maintain a principle always means optimism or it is valueless. The battle for progress requires faith, faith that through individual effort the world can, in degree, be made better, and this applies with full force to the development of any calling, such as ours, that has not quite outgrown the crudities of its origin.

It may be asked, and the question is a proper one, Why should the INTERNATIONAL DENTAL JOURNAL cease from its work and its influence and be lost, at this period in dentistry, where it is most needed? The answer must be that the eighteen years of experience has developed the fact that the dentists of the first decade of the twentieth century in America are not prepared to accept, much less stand, for the principles that this journal advocates. The tide of retrogression has been steadily flowing towards commercialism. The literature of dentistry to-day, in this country, is bound up in dollar journals and these constitute the intellectual pabulum of the large majority of the twenty-five thousand dentists of this country. It has been found a herculean task to meet this tendency, and while this Journal has not suffered seriously in the conflict, it was very evident that the end was not far distant when it would be forced to cease the struggle single-handed against this baleful influence that has become the dry-rot of the dental profession in America. In anticipation of this possibility, it was deemed wise, under the circumstances detailed in another article relative to the action of the National Dental Association, to close the life of this journal while still in full vigor.

The regret would be greater if it were not well understood that all reforms in the active life of the world must necessarily be slowly advanced, and the hope of the future in dentistry lies in a higher educated class. When these are in full activity a change will be manifest and the dollar will not then be the paramount motive for action.

The thanks of the Editor and his associates are due and are given freely to those who have stood faithfully by the INTERNATIONAL DENTAL JOURNAL through all these years. The societies that have, with very few breaks, furnished their proceedings freely and at much financial sacrifice, deserve special mention, for these contain the professional germ that must eventually give life and power to the entire body. To the New York Institute of Stomatology and its succession of able editors, this journal is deeply indebted and extends most grateful thanks for its disinterested and unselfish service. The same may be said of the American Academy of Dental Science and Harvard Odontological Society, both of Boston; the Academy of Stomatology of Philadelphia; the Section of Stomatology, American Medical Association, and to other organizations and individuals who have shown their interest through repeated contributions. To all of these and a still larger contingent who have quietly expressed their satisfaction with the journal and its aims, most cordial thanks and appreciation are extended.

The Editor is, and always has been, very cognizant of his limitations. He entered upon the duty in 1890 with many misgivings, and he now retires with the feeling that at no period in the work has he reached his ideal of what a dental periodical should be. He has the consciousness, however, that his aim has been to use every effort to urge the dental profession to stand for the highest. If that work has enabled any one to cultivate loftier aspirations he will feel that he has not used mental and physical strength in vain.

It is the natural result of the editorial position that the occupant of this chair will not always be understood and his motives will often be misconstrued. This has been the experience of the present writer. This must be counted among the unpleasant things to be left upon life's troubled pathway to be forgotten.

It is doubtful whether a journal such as the INTERNATIONAL will be established for many years to come. The men behind it who have sacrificed time, energy and money, are still in the front of the battle, but they feel, with the writer, that conditions must be totally changed before success will crown a similar effort.

The writer has no harsh word to express against our contemporaries, the so-called trade journals. Many evidences of good fellowship have been experienced from these, and while it is felt that their influence has been to the demoralization of the dental profession through the insidious poison of commercialism, they have lived up to, and been consistent with, business ideals and, in their way, have contributed to the practical knowledge of dentistry and thus indirectly have made it more perfect in its mechanical and scientific progress.

The writer has nothing to add by way of regrets. The retirement to a quieter life is anticipated with some pleasure. He has not reached the period when his voice and pen are to be silenced. Other avenues for both will be found, for his interest in his profession can be extinguished only when the last glimmer of life's sunset has ceased to fall upon his pathway.

Thus may we hope that in the years to come when the higher life of dentistry has been established, that the new era will usher in a living journal that will be the organ not only of a national society, but will represent the true aspirations of the entire dental fraternity of America. For the accomplishment of this let us all unite, for in its realization lives the hope that the dental profession of this country will attain the true professional standard recognized as such in all modern civilizations.

